# Granzyme A and CD160 expression delineates ILC1 with graded functions in the mouse liver

**DOI:** 10.1002/eji.202149209

**Published:** 2021-08-19

**Authors:** Chiara Di Censo, Marie Marotel, Irene Mattiola, Lena Müller, Gianluca Scarno, Giuseppe Pietropaolo, Giovanna Peruzzi, Mattia Laffranchi, Julija Mazej, Mohamed Shaad Hasim, Sara Asif, Eleonora Russo, Luana Tomaipitinca, Helena Stabile, Seung‐Hwan Lee, Laura Vian, Massimo Gadina, Angela Gismondi, Han‐Yu Shih, Yohei Mikami, Cristina Capuano, Giovanni Bernardini, Michael Bonelli, Silvano Sozzani, Andreas Diefenbach, Michele Ardolino, Angela Santoni, Giuseppe Sciumè

**Affiliations:** ^1^ Department of Molecular Medicine Sapienza University of Rome Laboratory affiliated to Istituto Pasteur Italia – Fondazione Cenci Bolognetti Rome Italy; ^2^ Ottawa Hospital Research Institute Cancer Therapeutics Program Ottawa Ontario Canada; ^3^ Centre for Infection, Immunity and Inflammation University of Ottawa Ottawa Ontario Canada; ^4^ Department of Biochemistry Microbiology, and Immunology University of Ottawa Ottawa Ontario Canada; ^5^ Laboratory of Innate Immunity Department of Microbiology Infectious Diseases and Immunology Campus Benjamin Franklin Charité‐Universitätsmedizin Berlin Berlin Germany; ^6^ Berlin Institute of Health (BIH) Anna‐Louisa‐Karsch Strasse 2 Berlin Germany; ^7^ Deutsches Rheuma‐Forschungszentrum Mucosal and Developmental Immunology Berlin Germany; ^8^ Division of Rheumatology Department of Internal Medicine III Medical University of Vienna Vienna Austria; ^9^ Center for Life Nano‐ & Neuro‐Science Fondazione Istituto Italiano di Tecnologia Rome Italy; ^10^ Translational Immunology Section Office of Science and Technology National Institute of Arthritis Musculoskeletal and Skin Diseases National Institutes of Health Bethesda Maryland USA; ^11^ Neuro‐Immune Regulome Unit National Eye Institute and National Institute of Neurological Disorders and Stroke National Institutes of Health Bethesda Maryland USA; ^12^ Division of Gastroenterology and Hepatology Department of Internal Medicine Keio University School of Medicine Tokyo Japan; ^13^ Department of Experimental Medicine Sapienza University of Rome Rome Italy; ^14^ IRCCS Neuromed Pozzilli Italy

**Keywords:** CD160, granzyme A, innate lymphoid cells, natural killer, Nfil3

## Abstract

Type 1 innate lymphoid cells (ILC1) are tissue‐resident lymphocytes that provide early protection against bacterial and viral infections. Discrete transcriptional states of ILC1 have been identified in homeostatic and pathological contexts. However, whether these states delineate ILC1 with different functional properties is not completely understood. Here, we show that liver ILC1 are heterogeneous for the expression of distinct effector molecules and surface receptors, including granzyme A (GzmA) and CD160, in mice. ILC1 expressing high levels of GzmA are enriched in the liver of adult mice, and represent the main hepatic ILC1 population at birth. However, the heterogeneity of GzmA and CD160 expression in hepatic ILC1 begins perinatally and increases with age. GzmA^+^ ILC1 differ from NK cells for the limited homeostatic requirements of JAK/STAT signals and the transcription factor *Nfil3*. Moreover, by employing *Rorc(γt)*‐fate map (fm) reporter mice, we established that ILC3‐ILC1 plasticity contributes to delineate the heterogeneity of liver ILC1, with RORγt‐fm^+^ cells skewed toward a GzmA^–^CD160^+^ phenotype. Finally, we showed that ILC1 defined by the expression of GzmA and CD160 are characterized by graded cytotoxic potential and ability to produce IFN‐γ. In conclusion, our findings help deconvoluting ILC1 heterogeneity and provide evidence for functional diversification of liver ILC1.

## Graphical abstract

Di Censo et al. identified distinct ILC1 populations in the mouse liver, defined by the expression of granzyme A (GzmA) and CD160. Expression of these molecules is finely tuned perinatally and delineates ILC1 with graded ability to produce IFN‐γ and to kill cancer cells. Moreover, ILC3‐ILC1 plasticity contributes to determining the heterogeneity of liver ILC1. These findings help deconvoluting phenotypic and functional heterogeneity of liver ILC1.

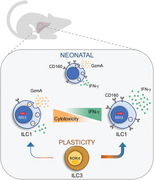

## Introduction

Type 1 innate lymphoid cells (ILC1) were identified in the liver and were originally considered as a phenotypically immature subset of natural killer (NK) cells, due to the expression of NK cell‐related markers [1]. Since then, ILC1 have been described in several lymphoid and non‐lymphoid organs, showing tissue‐specific properties only partially overlapping with NK cells [2, 3, 4]. ILC1 are generally defined by the ability to produce IFN‐γ and TNF‐α and for the requirement of the transcription factor T‐bet, while, differently from NK cells, ILC1 do not require Eomes expression [5, 6, 7, 8, 9]. Transcriptomic and genetic approaches have led to a refinement of the markers and factors determining the identity of liver ILC1, helping to discriminate these cells from NK cells, NKT cells, or even ILC1 in other tissues [8, 10, 11]. Liver ILC1 specifically relies on the transcription factor *Hobit* for development [12], while *Nfil3* deletion has limited impact on these cells compared to other ILCs [7, 13, 14, 15, 16, 17, 18]. These features place liver ILC1 on a distinct developmental trajectory in respect to NK cells and intestinal ILC1 [19].

Single‐cell RNA‐seq analyses have revealed wide transcriptional heterogeneity of ILC1 within the same tissue, both in mice and humans [11, 20, 21, 22, 23]. Distinct NK/ILC1 states have been identified in the mouse liver at steady state [24], and in pathological conditions (e.g., *Toxoplasma gondii* infection and cancer [11, 21]). Whereas single‐cell technology helped defining ILC1 subsets based on transcriptomic heterogeneity, our knowledge on whether these subsets differ for functional properties is still limited.

Here, we identified distinct ILC1 populations defined by the expression of granzyme A (GzmA) and CD160. GzmA^+^ ILC1 accumulated mainly in the liver, while were present at lower frequency in salivary glands and the lamina propria of the small and large intestine. ILC1 expressing both GzmA and CD160 represented the main hepatic ILC1 population at birth, while GzmA^+^CD160^–^ and GzmA^–^CD160^+^ ILC1 appeared perinatally. GzmA^+^ ILC1 differed from NK cells and NK cell derived‐ILC1‐like cells both at phenotypic level and for the requirements of JAK/STAT signaling and *Nfil3*. Moreover, we showed that ILC3‐ILC1 plasticity can also contribute to define the heterogeneity of hepatic ILC1, as part of liver ILC1 have lost RORγt expression, as observed by employing *Rorc(γt)*‐fate map (fm) reporter mice. Finally, we observed graded functions in ILC1 identified by GzmA and CD160, being CD160^+^ ILC1 better IFN‐γ‐producers, while less cytotoxic than CD160^–^ cells against tumor cells. These findings help to deconvolute ILC1 heterogeneity both at the phenotypic and functional level and to identify hepatic ILC1 skewed toward cytokine production or cytotoxicity.

## Results

### Liver ILC1 are heterogeneous for the expression of GzmA and CD160

To dissect the phenotypical heterogeneity of liver ILC1, we first mined single cell RNA‐seq data of hepatic NK1.1^+^NKp46^+^ cells [21] and defined 10 distinct transcriptional states (clusters 0–9 in Supporting Information Fig. S1A and Table S1). To discriminate ILC1 from NK cells and other ILCs, we applied signature module scores, obtained by Immgen, to the different clusters (Supporting Information Table S1). Cluster 2 was significantly enriched for ILC1 signature genes, while it was not associated with NK cell, ILC2, or ILC3 modules (Supporting Information Fig. S1B). Among the transcripts defining cluster 2, we found *Cd7*, *Cd160*, *Cxcr6*, *Cxcr3*, *Lag3*, and *Thy1* (encoding for CD90), which are conventionally associated to the ILC1 phenotype (Supporting Information Table S1).

**Figure 1:**
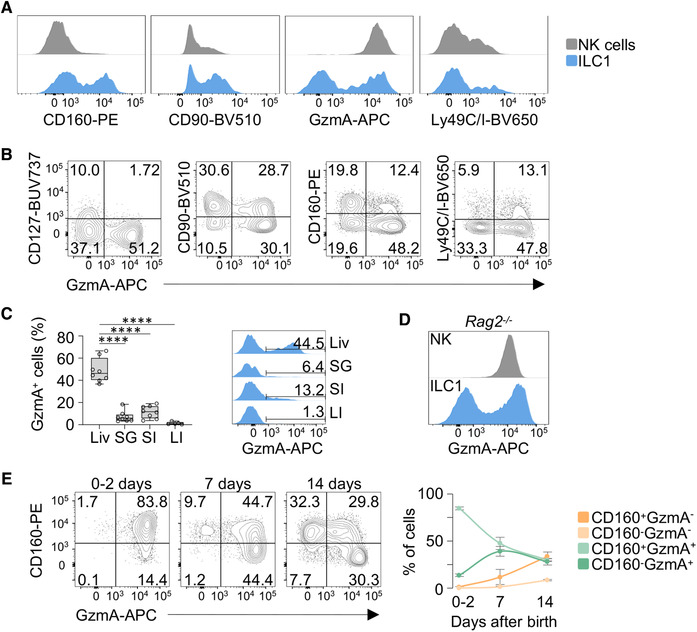
**GzmA^+^ ILC1 accumulate in the mouse liver. (A)** Histogram plots for the indicated markers are shown for liver NK cells (gray) and ILC1 (blue). Each marker was assessed by flow cytometry in at least 3 mice in three independent experiments. See Supporting Information S1A. **(B)** Representative contour plots show the expression of indicated markers versus GzmA in liver ILC1. **(C)** Box plot and histogram plot showing the percentage of GzmA^+^ cells among ILC1 isolated from liver (Li), salivary glands (SG), small intestine (SI) and large intestine (LI). Three independent experiments (2–3 mice per group) were performed (one‐way ANOVA was applied; ****P < 0.0001). **(D)** Histogram plot depicts GzmA expression in NK cells (gray) and ILC1 (blue) isolated from the liver of *Rag2^–/–^* mice. Data are representative of at least three independent experiments (*n* = 6). **(E)** Representative contour plots of CD160 and GzmA expression in ILC1 isolated from the liver of 0–2 days, 7 days and 14 days old mice. Scatter plot shows the percentage of the indicated populations at different ages. Data are shown as mean ± SD. Two independent experiments, with at least three mice per group, were performed.

Next, we dissected the distinct transcriptional states within cluster 2 and identified 163 genes differentially expressed in ILC1 (clusters a‐e), which included granzymes (*Gzma*, *Gzmb*), and several activating and inhibitory receptors (*Klra3, Klra4*, *Klra7‐9*) (Supporting Information Fig. S1C and Table S1). Having established these gene lists, we sought to screen the protein expression levels of an array of surface and effector molecules in hepatic ILC1, defined as Eomes^–^CD49a^+^ cells, by flow cytometry (see Supporting Information S1D for gating strategy). By employing this approach, we observed that the expression of CD160, CD90, GzmA, and Ly49C/I (encoded by *Klra3* and *Klra9*, respectively) delineated discrete ILC1 populations (Fig. 1A; Supporting Information S1E).

*Gzma* was one of the most heterogeneous transcripts among the ILC1 clusters, and its protein expression identified a population characterized by low levels of CD127, CD90, and high levels of Ly49C/I (Fig. 1B and Supporting Information S1F). Further analysis of tissues enriched in type 1 ILCs, such as salivary glands, and the lamina propria of the small and large intestine, revealed that GzmA^+^ ILC1 accumulated mainly in the liver (Fig. 1C; Supporting Information S1G). Moreover, development of GzmA^+^ ILC1 also occurred in *Rag2^–/–^* mice, confirming that these cells did not represent a contamination of liver resident adaptive T cells (Fig. 1D).

Given that the appearance of hepatic ILC1 and NK cells is dynamically regulated during mouse ontogeny, we sought to evaluate whether the ILC1 phenotype was characterized by changes in the GzmA and CD160 expression pattern during neonatal and perinatal life. By tracking these markers in the first 2 weeks after birth, we found that neonatal ILC1 mainly comprised CD160^+^GzmA^+^ ILC1, while in the perinatal life CD160^–^GzmA^+^ cells appeared earlier than CD160^+^GzmA^–^ and CD160^–^GzmA^–^ cells (Fig. 1E). Altogether, our data provide evidence for the phenotypic heterogeneity of liver ILC1 based on the expression of both surface receptors and effector molecules. In particular, we identified a population of ILC1 expressing high levels of GzmA, which is mainly found in the liver. Moreover, we show that CD160 and GzmA expression is finely tuned during mouse ontogeny.

### Lineage relationships of liver ILC1 populations

**Figure 2:**
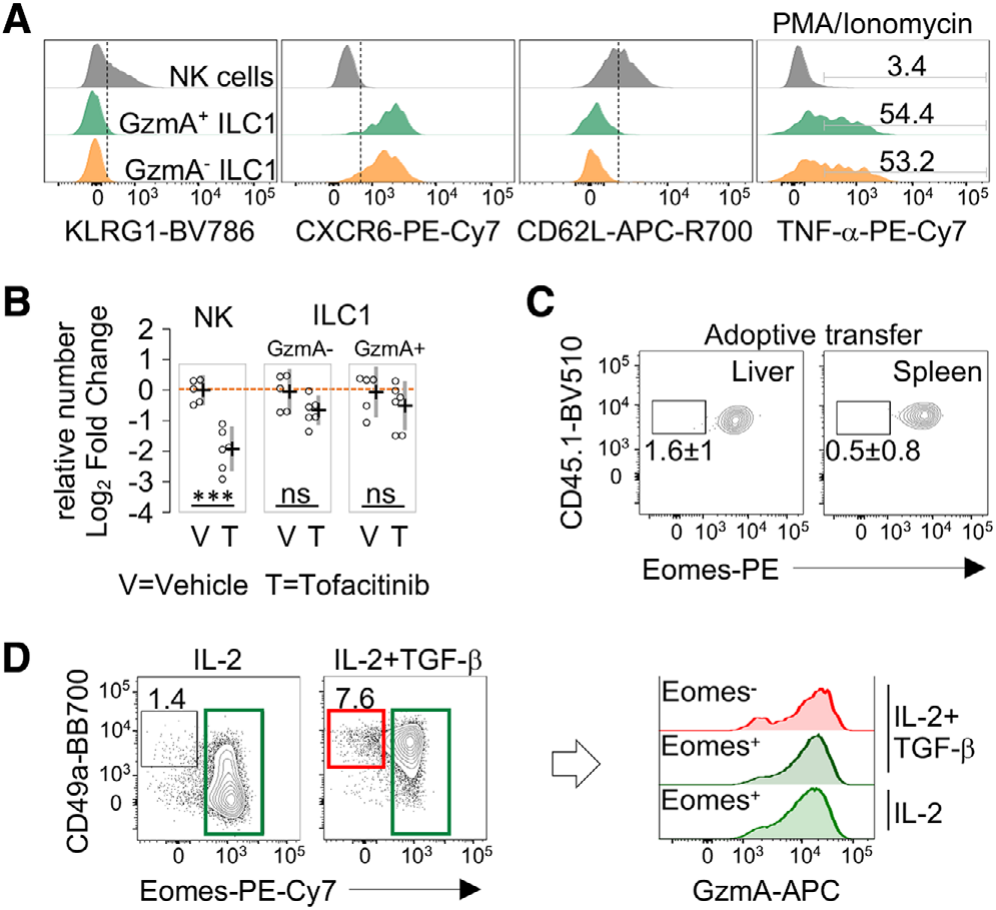
**NK cells have limited potential to give rise to GzmA^+^ ILC1. (A)** Flow cytometry stacked histogram plots for KLRG1, CXCR6, and CD62L in freshly isolated hepatic NK cells (gray), GzmA^+^ ILC1 (green) and GzmA^–^ ILC1 (orange); and TNF‐α expression after PMA/Ionomycin stimulation. A representative experiment (3 mice per group), of at least three performed, is shown. **(B)** Scatter plot displays the relative number of NK cells, GzmA^+^ and GzmA^–^ ILC1 in tofacitinib‐ (*n* = 6) and vehicle‐treated (*n* = 5) mice (as log_2_ fold change, FC relative to control mice). Mean with 95% CI is shown. Each dot represents an individual mouse. Two independent experiments were combined (one‐way ANOVA was applied; ***P < 0.001; ns, not significant). **(C)** Contour plots show percentage of donor CD45.1^+^Eomes^–^ NK cells, isolated from the spleen and liver of recipient mice, 2 weeks post‐injection. Data represent one experiment of out of four performed (*n* = 4). **(D)** Representative contour plots of CD49a and Eomes expression in splenic NK cells cultured with IL‐2 or IL‐2/TGF‐β for 5–7 days (left). Histogram plots (right) display GzmA expression in the indicated conditions. Data shown are representative of three independent experiments (*n* = 3).

The identification of ILC1 expressing high levels of GzmA raised the question as to whether this population share features with NK cells in terms of phenotype, homeostatic requirements, and differentiation. The CXCR6^+^CD62L^–^ profile and the lack of KLRG1 expression observed in GzmA^+^ ILC1 (Fig. 2A) indicated that these cells phenotypically resembled conventional tissue‐resident ILC1 rather than mature NK cells or NK cell‐derived ILC1. In line with this observation, both GzmA^–^ and GzmA^+^ ILC1 expressed high levels of TNF‐α when stimulated with PMA/Ionomycin (Fig. 2A, right panel). Since the homeostatic number of liver NK cells is highly affected upon interference with the JAK/STAT signaling pathway [25], we sought to define the impact of JAK inhibition on ILC1 populations. Therefore, we employed a pharmacological approach consisting of oral administration of tofacitinib, a pan‐JAK inhibitor, for 7 days, and used *Rag2^–/–^* mice to exclude competition for cytokine availability between innate and adaptive lymphocytes. In contrast to what observed for NK cells, tofacitinib administration only minimally reduced the number of GzmA^+^ ILC1 (Fig. 2B, absolute numbers in Supporting Information S2A), implying that these cells had distinct requirements for JAK signals from NK cells and supporting the hypothesis that NK cells have a limited potential to give rise to GzmA^+^ ILC1 in homeostatic conditions. Consistent with these findings and previous observations [1, 8, 21], splenic CD45.1^+^ NK cells stably expressed Eomes up to 2 weeks after adoptive transfer in *Rag2^–/–^* mice (Fig. 2C; Supporting Information S2B).

To further assess whether NK cells could trans‐differentiate in GzmA^+^ ILC1, we took advantage of a TGF‐β‐based culture system allowing to recapitulate in vitro the NK/ILC1 transition [26, 27]. The treatment with TGF‐β induced CD49a expression in NK cells, while reduced the levels of perforin and Eomes, as compared to cells treated with IL‐2 only (Supporting Information S2C). In this setting, TGF‐β led to the differentiation of cells expressing CD49a and low or no Eomes (<10%), which still retained high levels of GzmA (Fig. 2D). These data suggest that, in microenvironments enriched in TGF‐β, NK cells could become a source of Eomes^–^GzmA^+^ ILC1.

**Figure 3:**
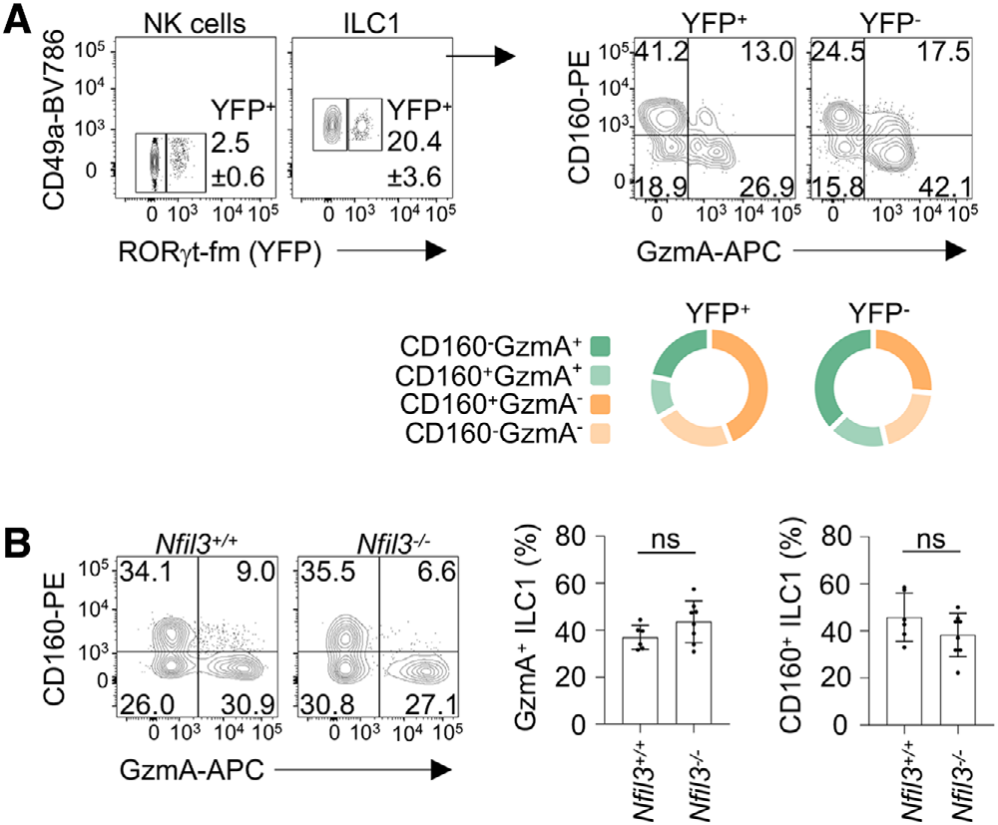
**Contribution of ILC3‐ILC1 plasticity and *Nfil3* in the establishment of liver ILC1 heterogeneity. (A)** Contour plots depict the percentage of YFP^+^ cells among NK cells and ILC1 isolated from livers of RORγt‐fm mice (left). Contour plots and donut charts (right) show the proportions of ILC1 discriminated by CD160 and GzmA expression, within YFP^+^ and YFP^–^ cells. Two independent experiments were performed (*n* = 6). **(B)** Representative contour plots of CD160 and GzmA expression in ILC1 isolated from liver of *Nfil3^+/+^* (*n* = 6) and *Nfil3^–/–^* (*n* = 8) mice. Histograms show the percentages of total GzmA^+^ and CD160^+^ ILC1 in *Nfil3^+/+^* and *Nfil3^–/‐^* mice. Data are shown as mean ± SD (two‐tailed Student's *t*‐test was applied; ns, not significant). Three independent experiments were combined.

Along with NK cells, ILC3 can also trans‐differentiate to ILC1 in the intestine and other tissues, giving rise to the so called “ex‐ILC3” [3, 7, 28, 29, 30, 31]. To evaluate the contribution of ILC3 plasticity in the establishment of distinct hepatic ILC1 populations, we employed *Rorc(γt)*‐fate map (fm) reporter mice [29]. We found that RORγt‐fm^+^ cells ranged from 15 to 25% within the hepatic ILC1 population; in contrast, these cells represented less than 5% of the total NK cells (Fig. 3A, left panels). We also observed that RORγt‐fm^+^ cells were able to give rise to the whole spectrum of hepatic ILC1, according to GzmA and CD160 expression (Fig. 3A, right panels). However, RORγt‐fm^+^ cells were enriched in ILC1 showing a CD160^+^GzmA^–^ phenotype (Fig. 3A; Supporting Information S2D).

*Nfil3* deletion leads to a wide spectrum of developmental defects in innate lymphocytes, with the number of NK cells resulting highly reduced in several tissues, except for salivary glands [3, 13, 14, 19]. Distinct tissue‐resident ILC1 have a differential requirement for *Nfil3*, being intestinal CD160^+^ ILC1 more affected as compared to liver ILC1 [3, 7, 14, 15, 16, 17, 18, 19]. In line with previous findings, we found that deletion of *Nfil3* had a higher impact on liver NK cells, as compared to ILC1 (Supporting Information S2E‐F). On the other hand, the relative frequencies of CD160^+^ and GzmA^+^ within the residual *Nfil3^–/–^* ILC1 were not significantly altered compared with wild‐type cells (Fig. 3B), indicating that the liver ILC1 defined by these two markers had overlapping requirements for *Nfil3*.

Collectively, these data support the conclusion that GzmA^+^ ILC1 differ from NK cells, and represent a distinct state of ILC1. Our findings also suggest that plasticity of ILC3 can contribute to delineate the heterogeneity of hepatic ILC1.

### Liver ILC1 subsets show graded cytotoxic potential and ability to produce IFN‐γ

**Figure 4:**
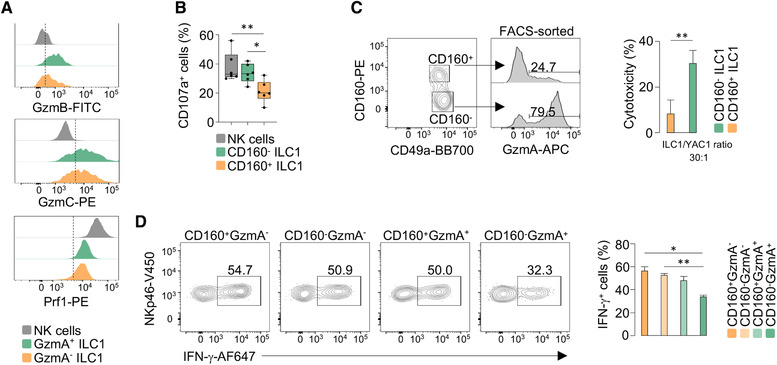
**Graded functionality of liver ILC1. (A)** Stacked histogram plots display the expression of the indicated markers for liver NK cells (gray), GzmA+ ILC1 (green) and GzmA^–^ ILC1 (orange). Each marker was assessed by flow cytometry in at least three mice in three independent experiments. **(B)** Box plot shows the percentage of CD107a^+^ cells in NK cells, CD160^–^ ILC1 and CD160^+^ ILC1, stimulated with plate bound anti‐NKR‐P1C. Data are presented as mean ± SD (one‐way ANOVA was applied; *P < 0.05; **P < 0.01). Three independent experiments were combined (*n* = 7). **(C)** FACS plots of GzmA expression of FACS‐sorted CD160^+^ and CD160^–^ hepatic ILC1. Histogram plot displays the percentage of cytotoxicity of CD160^+^ and CD160^–^ ILC1 against YAC‐1 cells, at an effector:target ratio of 30:1. Data are shown as mean ± SD of four independent experiments (two‐tailed Student's *t*‐test was applied; **P < 0.01). **(D)** Flow cytometry contour plots depict IFN‐γ expression in indicated ILC1 populations stimulated with IL‐12 plus IL‐18 for 6 h. Histogram plot shows percentage of IFN‐γ in ILC1 subsets. Data are presented as mean ± SD (one‐way ANOVA was applied; *P < 0.05; **P < 0.01) (*n* = 3). Four independent experiments were performed.

To begin to assess the functional properties of ILC1 populations, we evaluated the expression of cytotoxic mediators in GzmA^+^ and GzmA^–^ ILC1. Both populations were characterized by perforin expression and high levels of GzmC, while GzmB was expressed at higher levels in GzmA^+^ cells (Fig. 4A; Supporting Information S3A), suggesting distinct cytotoxic potential within the ILC1 population. Next, we employed ex vivo assays to determine the cytotoxic potential of ILC1. First, we stimulated lymphocytes from the murine liver with plate‐bound anti‐NKR‐P1C. Both NK cells and ILC1 presented surface display of the degranulation marker CD107a/LAMP‐1 (Supporting Information S3B). Moreover, degranulation resulted in a marked reduction of intracellular GzmA in NK cells and ILC1 (Supporting Information S3C), which limited our ability to use GzmA to dissect ILC1 populations. However, we observed that, after anti‐NKR‐P1C stimulation, the surface expression of CD107a was lower in CD160^+^ ILC1 than CD160^–^ ILC1 (Fig. 4B). We next analyzed the cytotoxic activity of FACS‐sorted CD160^+^ and CD160^–^ ILC1 against YAC‐1 cells. Sorted CD160^–^ were enriched in GzmA expression and showed a significantly higher cytotoxicity compared with CD160^+^ ILC1 (Fig. 4C). Altogether, these observations provide evidence for differential ability to mediate granule‐dependent killing in liver ILC1.

Lymphocyte populations often show graded potential for cytokine production [23]; thus, we hypothesized that ILC1 discriminated by CD160 and GzmA would also differ in their ability to produce IFN‐γ in response to cytokines. Upon stimulation with IL‐12 plus IL‐18, we found that the distinct ILC1 populations showed a graded ability to produce IFN‐γ, with CD160^–^GzmA^+^ cells showing lower capacity to produce this cytokine, compared with other ILC1 populations (Fig. 4D). These findings provide evidence for the distinct ability of liver ILC1, not only to mediate granule‐dependent killing, but also to produce IFN‐γ.

## Discussion

Our study dissects the phenotypical and functional heterogeneity of liver ILC1 subsets by combining transcriptomic and flow cytometry approaches, together with functional assays. First, we identified an array of proteins differentially expressed in hepatic ILC1, including effector molecules and surface receptors, helping to establish discrete populations. Next, based on the expression of GzmA and CD160, we provide evidence for a phenotypical and functional continuum of hepatic ILC1 endowed with graded ability to produce IFN‐γ in response to cytokines and to kill target cells. This is relevant in the context of viral infection, since recent findings have shown that ILC1 confer early protection in sites of viral entry by rapidly producing IFN‐γ in response to IL‐12 [32]. As well, ILC1 can provide memory‐like responses either against mouse CMV (MCMV) [33] or in response to haptens [34, 35]. Interestingly, IL‐7R is upregulated in memory ILC1 in both conditions, which could underly changes in the expression of this receptor and/or specific expansion of IL‐7R^+^ ILC1 during memory formation. Moreover, the identification of ILC1 expressing high levels of granzymes, along with perforin, could be also relevant in the context of liver physiology, helping to maintain tissue integrity, as well as pathology, including cancer. These observations open toward distinct modalities for liver ILC1 to contribute to the immune response involving not only production of cytokines, but also the potential to directly eliminate target cells.

Our study also reveals that the appearance of hepatic ILC1, differing for GzmA and CD160 expression, is temporally distinct. At birth, CD160^+^GzmA^+^ ILC1 are the main hepatic ILC1 population, representing the only ILC population with cytotoxic potential in the mouse liver, due to the delayed appearance of NK cells. However, within the first 2 weeks after birth, the hepatic ILC1 acquire the CD160/GzmA expression pattern found in young/adult mice, suggesting that both differentiation of liver neonatal ILC1 and de novo generation by ILC1 progenitors could participate in the establishment of the ILC1 heterogeneity observed in the adult. Further complexity is provided by evidence showing that liver ILC1 can develop in an IFN‐γ‐dependent manner [1, 36], indicating that distinct cytokines might be responsible for the regulation of the homeostatic pool of ILC1 populations. Moreover, we observed that ILC3‐ILC1 plasticity could also contribute to the generation of ILC1 heterogeneity, since ex‐ILC3 can account up to 20% of the total hepatic ILC1. Thus, liver ILC1 might represent a spectrum of cells established via distinct development and plasticity events.

Looking forward, dissecting the functional diversity of the different ILC1 populations might be critical for the design of therapeutic strategies aimed at potentiating both antiviral and anticancer responses in innate lymphocytes.

## Materials and methods

### Mice

Female C57BL/6J, CD45.1 and *Rag2^–/–^* (Jackson mouse stock no. 008449) mice were purchased from Charles River. *Nfil3^–/‐^* mice (obtained by Prof. Brady [13]) were housed in the animal facility at Medical University of Vienna; *Nfil3^–/–^ and Nfil3^+/+^* littermates were used. *Rorc(γt) Cre* × *ROSA26‐YFP* (RORγt‐fm reporter mice) were generated as described [29], and housed in individually ventilated cages under specific pathogen‐free conditions. All the experiments with young mice were performed using 6–10 weeks old mice. Neonatal and perinatal mice were analyzed from birth to the first 2 weeks of age. Tofacitinib (30 mg/kg) or vehicle (control) were administered orally twice daily for 7 days as previously described [37].

### Single cell RNA‐seq analysis

The R package Seurat version 4.0.1 was used under RStudio version 4.0.2 for data trimming, unsupervised clustering, and visualization according to the authors’ guidelines [38]. We performed unbiased clustering of liver NK1.1^+^NKp46^+^ cells using a previously published transcriptomic study [21], referring to the WT uninfected liver sample, with the accession number GSE124577. The code utilized to run the transcriptomic analysis is available at https://github.com/ML1990‐Lab/Di‐Censo‐et‐al.‐2021. Detailed description in Supporting Information S1.

### Cell isolation from tissues and FACS

Cells from the liver and spleen were obtained as previously described [37]. Cells from intestine lamina propria and salivary glands were isolated after incubation with 0.5 mg/mL DNase I and 0.25 mg/mL Liberase TL (Roche) [39]. Gating and sorting strategies are provided in Supporting Information S1D and 2B. The complete list of antibodies is provided in Supporting Information Table S1. Samples were acquired using a FACSCantoII or an LSRFortessa (BD Biosciences). Sorting was performed using a FACSAriaIII (BD Biosciences). Data were analyzed with FlowJo version 10 software (Tree Star). The authors adhered to the guidelines for the use of flow cytometry and cell sorting in immunological studies [40].

### Ex vivo assays

For intracellular cytokine detection, cells were treated with Cell Stimulation Cocktail (ThermoFisher), containing PMA/Ionomycin, in RPMI‐1640 complete medium (supplemented with 10% FBS [ThermoFisher], penicillin and streptomycin [100 U/mL, 100 μg/mL, Euroclone], and l‐glutamine [2 mM, Euroclone]), in the presence of GolgiStop (BD Biosciences) for 2 h at 37°C. Alternatively, cells were stimulated with IL‐12 (100 ng/mL) plus IL‐18 (100 ng/mL) for 4–6 h in the presence of GolgiStop and Brefeldin A (Sigma). For CD107a detection, flat‐bottom high‐binding 96‐well plates were coated with 5 μg of anti‐NKR‐P1C (BioXCells) in each well overnight at 4°C. Lymphocytes enriched from the murine liver were plated in complete medium supplemented with IL‐2 (200 UI/mL) and GolgiStop (BD Biosciences) and incubated for 4 h. Fluorochrome‐conjugated CD107a antibody was added during the last 2 h. For the in vitro NK/ILC1 transition experiment, cells were treated with IL‐2 (200 UI/mL) and IL‐2 plus TGF‐β (10 ng/mL, Peprotech) for 5–7 days. The flow cytometry‐based killing assay was performed as previously described [41]. Briefly, 10 livers were pooled and CD160^+^ and CD160^–^ ILC1 were sorted and incubated with CFSE‐labeled target cells (YAC‐1) at 30:1 effector/target (E/T) ratio, for 4 h. CFSE‐labeled cells alone were used as control. Cells were washed and 20.000 APC‐labeled microbeads (Spherotech) were added to each sample to allow calculation of specific lysis.

### Statistical analysis

Student's *t*‐test and ANOVA were used to quantify statistical deviation between experimental groups, as indicated in figure legends. Asterisks denote significant differences. *P < 0.05; **P < 0.01; ***P < 0.001; ****P < 0.0001.

## Ethics approval statement for animal studies

All animal studies were approved by the Italian Ministry of Health (authorization numbers 255/2018‐PR and 727/2019‐PR) and performed in compliance with European animal welfare regulations and Canadian Institutes of Health Research.

## Conflict of Interest

The authors declare no conflict of interest.

## Author contribution

C.D.C contributed to experimental design, performed, analyzed and interpreted all the experiments; wrote the manuscript. M.M., I.M., L.M. G.Sca., G.Pi., G.Pe., J.M., M.S.H., S.A., E.R., L.T., H.S., S.‐H.L., L.V., Y.M., H.‐Y.S, C.C. helped with performing experiments. M.L. performed scRNA‐seq analysis. M.G., A.G., G.B., M.B., S.S., A.D., M.A., A.S. and G.Sci. contributed to the experimental design and data interpretation, made helpful suggestions and helped to write the manuscript. GSci designed and interpreted all the experiments, conceived the project and wrote the manuscript.

### Peer review

The peer review history for this article is available at https://publons.com/publon/10.1002/eji.202149209

**Abbreviations**
IFN: interferon
IL: interleukin
ILC: innate lymphoid cell
NCR: natural cytotoxicity triggering receptor
NK: natural killer
TNF: tumor necrosis factor.

## Supporting information

Supporting information

Supporting information

## Data Availability

The data that support the findings of this study are available from the corresponding author upon reasonable request.
