# Supplementary material for: Granzyme A and CD160 expression delineates ILC1 with graded functions in the mouse liver
Source: Eur J Immunol. 2021 Aug 19;51(11):2568–75. doi: 10.1002/eji.202149209 (PMC9292164; doi:10.1002/eji.202149209)
Supplement: Supplementary file 2 — Supporting information [file EJI-51-2568-s001.pdf]

Supporting Information 1

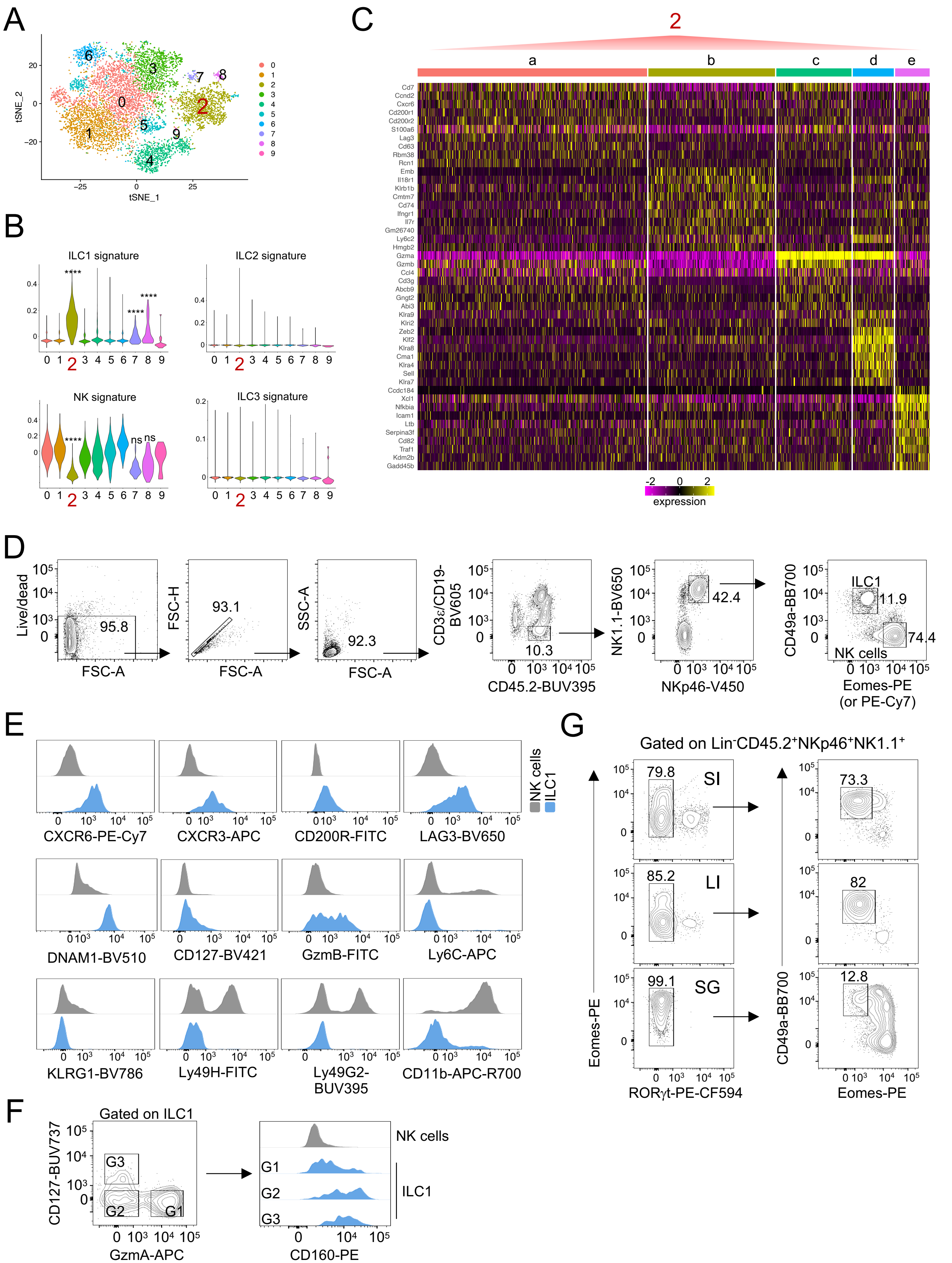

Supporting Information 1.

(A) t-SNE plot of 9,073 cells revealing 10 distinct clusters (from 0 to 9). Each different color in the legend identifies a specific cluster. Cluster 2 defining ILC1 is highlighted. In details, in order to remove poor quality cells, doublets and stressed cells, we removed those with a gene number less than 200, or higher than 2500 [42]. Mitochondrial gene ratio was calculated to filter out cells with more than 10% of gene contribution from mitochondrial genes. The resulting dataset was further processed with "SCTransform" for normalization and data scaling. In addition, mitochondrial and cell cycle genes were regressed out [43]. Highly variable genes (HVG, n=3000) were also identified with the "SCTransform" function. The HVGs were used as input for principal component analysis (PCA). The first 20 PCAs were utilized in the subsequent analysis. With a resolution of 0.6, unsupervised graph-based clustering was performed by the function "FindCluster". Cells were then embedded by t-SNE plot. To assign cell identities, we applied the "FindAllMarkers" to identify differentially expressed genes (DEGs) among all genes by using Wilcoxon rank sum test. We selected only genes that showed 1) a minimal expression (min.pct ≥ 25%) in at least one cluster and 2) an adjusted p-value ≤ 0.05.

(B) Violin plots represent cluster specific gene signature (one-way ANOVA with Dunnett's multiple comparisons test was applied; ****, P<0.0001; ns, not significant). For the definition of cell identity, dedicated immune signature module scores were added to each cell by the "AddModuleScore" function according to specific lists of known cell type markers deriving from the Immunological Genome Project (Immgen) tool "population comparison" (http://rstats.immgen.org/PopulationComparison/index.html). In details, the mouse Microarray dataset V2 was explored to identify unique genes for NK, ILC1, ILC2 and ILC3, by comparing multiple populations by ratio of expression means.

(C) Heatmap of the top 10 marker genes, tested with a Wilcoxon rank sum test, which better divided ILC1 into clusters. Five ILC1 transcriptional states (a, b, c d, e) were obtained by repeating the Seurat protocol, using for cell clustering 15 PCA and a resolution of 0.8.

(D) Gating strategy for the characterization of liver NK cells and ILC1. FSC-H, forward scatter height; FSC-A, forward scatter area; SSC-A, side scatter area. After dead cells and doublets exclusion, lymphocytes were electronically gated based on FSC/SSC parameters and then gated on $CD3\varepsilon^{-}CD19^{-}CD45.2^{+}$ population. Next, NK cells and ILC1 were identified as $NK1.1^{+}NKp46^{+}$ cells and finally separated according to CD49a and Eomes expression. For intracellular staining, cells were fixed and permeabilized using the FoxP3/Transcription Factor Staining Set (eBioscience) according to the manufacturer's instructions. Cytofix/Cytoperm Fixation/Permeabilization Solution Kit (BD Biosciences) was used to evaluate cytokine expression.

(E) Histogram plots show the expression of indicated markers in liver NK cells (gray) and ILC1 (blue). Each marker was assessed by flow cytometry in at least 3 mice in three independent experiments.

(F) Representative contour plot of CD127 and GzmA expression in liver ILC1 populations (G1, G2, G3) and histograms of CD160 expression in NK cells (gray) and G1, G2 and G3 populations. Data are representative of three independent experiments (n=5).

(G) Representative contour plots of the expression of Eomes and RORγt and CD49a and Eomes in $Lin(CD3\varepsilon CD19)^{-}CD45.2^{+}NKp46^{+}NK1.1^{+}$ cells isolated from small intestine, liver and salivary glands.

Supporting Information 2

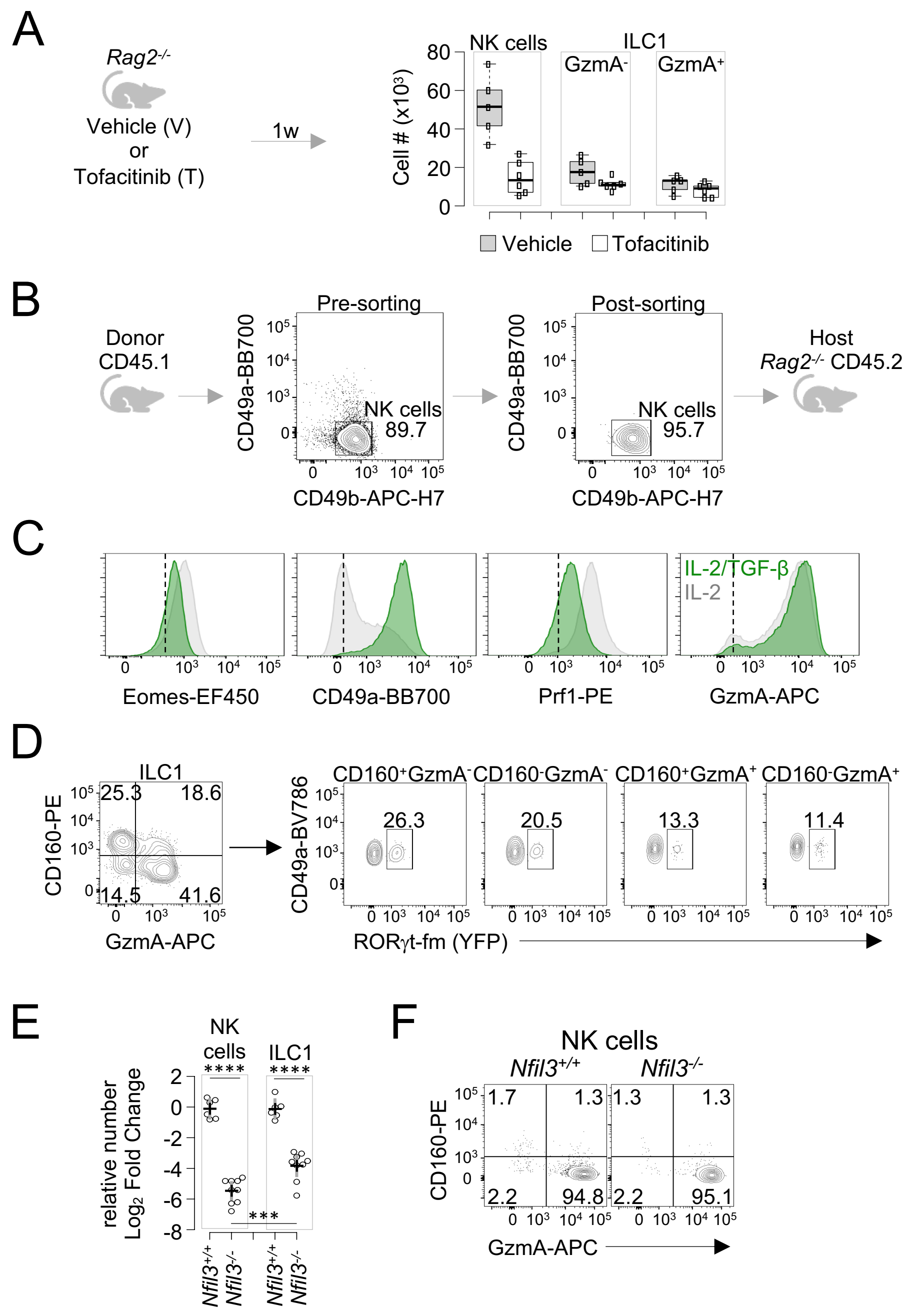

Supporting Information 2.

(A) Mice were administered with tofacitinib (n=6) or vehicle (n=5), twice daily for 7 days. Box plots show the absolute number of liver NK cells, GzmA$^-$ ILC1 and GzmA$^+$ ILC1. Each dot represents an individual mouse. Two independent experiments were combined.

(B) Schematic representation of adoptive transfer experiment. Splenic NK cells from CD45.1 mice were enriched by NK cell Isolation Kit, mouse (Miltenyi Biotec) and, then, sorted according to the indicated gating strategy. To reduce cellular stress, cells were sorted in gentle conditions using a ceramic nozzle of size 100 µm, a low sheath pressure of 19.84 pound-force per square inch (psi) that maintains the sample pressure at 18.96 psi and a maximum acquisition rate of 1500 events/s. Following isolation, an aliquot of each tube of the sorted cells was evaluated for purity at the same instrument resulting in an enrichment >95% for each sample. Next, $5x10^5$ NK cells were transferred into *Rag2*$^{-/-}$ recipient mice (CD45.2) via tail vein injection and analyzed from spleen and liver after 2 weeks.

(C) Representative histogram plots for CD49a, Eomes, Prf1 and GzmA expression in sorted NK cells cultured with IL-2 only and IL-2 + TGF-β for 5-7 days. Data shown are representative of three independent experiments (n=3).

(D) Contour plots showing the expression of CD160 and GzmA in liver ILC1 and the percentage of YFP$^+$ cells among the indicated population of ILC1 of RORγt-fm reporter mice. Two independent experiments (n=6) were performed.

(E) Scatter plot displays the relative number of liver NK cells and ILC1 in *Nfil3*$^{+/+}$ (n=6) and *Nfil3*$^{-/-}$ (n=8) mice (as log2 fold change, FC relative to control mice). Mean with 95% CI is shown. Each dot represents an individual mouse. Three independent experiments were combined (one-way ANOVA was applied; ***, P<0.001; ****, P<0.0001).

(F) Representative contour plots of CD160 and GzmA expression in NK cells isolated from liver of *Nfil3*$^{+/+}$ (n=6) and *Nfil3*$^{-/-}$ (n=8) mice. Three independent experiments were performed.

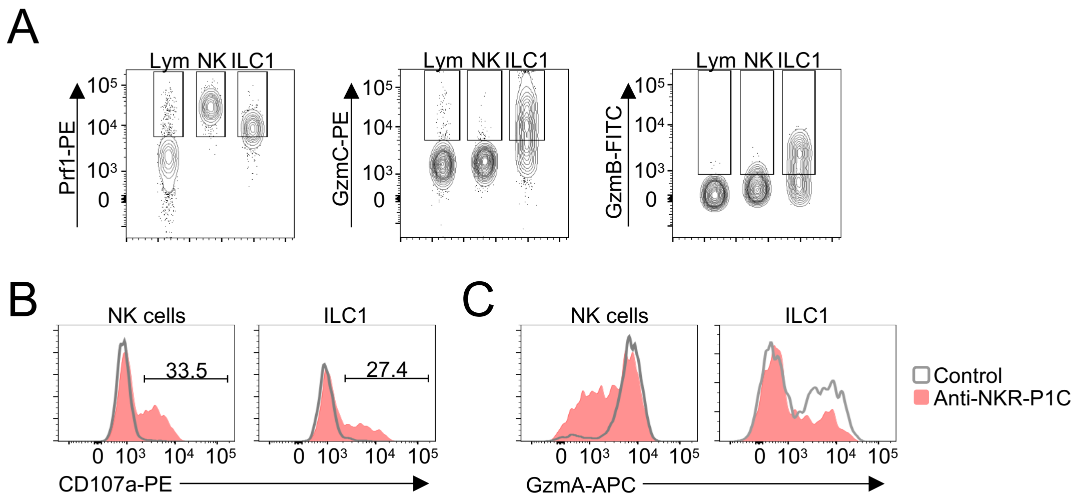

Supporting Information 3.
(A) Representative contour plots of Prf1, GzmC and GzmB expression in total lymphocytes (Lym), NK cells and ILC1 (concatenated). Each marker was assessed by flow cytometry in at least 3 mice in three independent experiments.
(B, C) Flow cytometry histogram plots show CD107a **(B)** and GzmA **(C)** expression in liver NK cells and ILC1 stimulated with plate bound anti-NKR-P1C (light red) and controls (gray). $2x10^5$ cells were plated. A representative experiment of three performed is shown (n=6).

Supporting Table 1.
Supporting Table 1 contains the gene signatures utilized to identify ILC1 defined by Immgen; the differentially expressed genes as defined in **Supporting Information 1A**; the differentially expressed genes as defined in **Supporting Information 1C**; the list of antibodies used.
